# The Effects of Negative Pressure Induced by Flow Separation Vortices on Vocal Fold Dynamics during Voice Production

**DOI:** 10.3390/bioengineering10101215

**Published:** 2023-10-18

**Authors:** Weili Jiang, Xudong Zheng, Charles Farbos de Luzan, Liran Oren, Ephraim Gutmark, Qian Xue

**Affiliations:** 1 Mechanical Engineering Department, Rochester Institute of Technology, Rochester, NY 14623, USA; wxjeme@rit.edu (W.J.); xxzeme@rit.edu (X.Z.); 2 Mechanical Engineering Department, University of Maine, Orono, ME 04469, USA; 3 Department of Otolaryngology Head and Neck Surgery, University of Cincinnati, Cincinnati, OH 45267, USA; farboscs@ucmail.uc.edu (C.F.d.L.); orenl@ucmail.uc.edu (L.O.); 4 Department of Aerospace Engineering and Engineering Mechanics, University of Cincinnati, Cincinnati, OH 45267, USA; gutmarej@ucmail.uc.edu

**Keywords:** flow separation vortices, vocal fold, intraglottal negative pressure

## Abstract

This study used a two-dimensional flow-structure-interaction computer model to investigate the effects of flow-separation-vortex-induced negative pressure on vocal fold vibration and flow dynamics during vocal fold vibration. The study found that negative pressure induced by flow separation vortices enhances vocal fold vibration by increasing aeroelastic energy transfer during vibration. The result showed that the intraglottal pressure was predominantly negative after flow separation before gradually recovering to zero at the glottis exit. When the negative pressure was removed, the vibration amplitude and flow rate were reduced by up to 20%, and the closing speed, flow skewness quotient, and maximum flow declination rate were reduced by up to 40%. The study provides insights into the complex interactions between flow dynamics, vocal fold vibration, and energy transfer during voice production.

## 1. Introduction

The production of the human voice is a complex process characterized by the finely tuned vibration of the true vocal fold pair within the larynx. This mechanism transforms a continuous stream of respiratory air into a pulsating airflow, forming the primary sound source of the voice. The larynx also houses a pair of false vocal folds, which are situated just above the true vocal folds. While the false vocal folds generally have a minimal role in voice production, they have the potential to positively contribute to sound intensity [1] and be engaged in certain types of voice production [2]. One of the important goals in voice production research is to understand the fundamental mechanisms that govern the intricate interactions among glottal aerodynamics, tissue biomechanics, vibratory dynamics, and acoustics. The improved understanding can provide scientific insights into the management of voice health.

Considerable research has focused on the development of intraglottal pressure during the closing phase of vocal fold vibrations. This process holds significance because, during this period, the glottis forms a divergent shape that can cause flow separation and complex pressure forces on the vocal fold surfaces impacting vibration and flow dynamics. For instance, in experiments using excised canine larynges, Oren et el. [3] found a positive correlation between the intraglottal negative pressure and the sound pressure level. Moreover, vortical structures and the associated flow turbulence generated from the flow separation process were found to affect quadrupole sound source, characterized by their broad spectral range and high frequencies [4,5,6], and vocal fold vibration [3,7,8,9]. Research has shown that flow separation occurs when the maximum cross-sectional area is one to two times the minimum glottal opening area [10,11]. As airflow passes this point, a circulating flow area forms between the jet and the medial glottal wall. Within this area, the intraglottal pressure is negative (gauge pressure) and it gradually recovers to atmospheric pressure (zero gauge pressure) at the glottal exit. Studies have observed that the lowest intraglottal pressure ranges between −0.5 and −0.2 times the subglottal pressure (gauge pressure) in the absence of the supraglottal tract [9,12,13,14,15], and between −0.5 and −1.2 times the subglottal pressure (gauge pressure) when the supraglottal tract is present [16,17].

In an experimental study involving excised canine larynges [7], it was shown that vortices form near the superior aspect of the folds after flow separation. These flow separation vortices (FSV) induce increased negative pressure at the superior aspect of the folds. Later experimental and computational studies also showed that the strength of FSV, and subsequently the negative pressure they augment, are proportional to the magnitude of the glottal divergent angle [3,8]. Farbos de Luzan et al. [18] quantified the intraglottal negative pressure induced by FSV using large eddy simulation by comparing the pressure fields between a divergent channel and a straight channel. The geometric model is static but with a time-varying pressure waveform applied at the inlet of the domain. They suggested that FSV in the divergent section of the true vocal folds was responsible for 136% more pressure reduction during vocal fold closing. Sundström et al. [8] investigated the impact of flow-separation-vortex-induced negative pressure (FSV_NP_) on the glottal dynamics using flow-structure-interaction (FSI) modeling. They reported that the FSV produced strong negative pressure on the folds, which was correlated with the vortical strength, and that the aerodynamic force induced by the FSV was at times higher than the elastic recoil force in the tissues. The increased negative pressure induced by FSV leads to a hypothesis that FSV can exert an additional pulling force on the vocal fold during closing and increase the flow deceleration rate. Notably, the rapid deceleration of airflow during the closing phase, quantified by MFDR, has been shown to have strong correlations with sound pressure level (SPL), acoustic energy in higher harmonics, and vocal efficiency loudness [19,20,21].

Much research effort has been dedicated to examining the effect of flow separation on glottal dynamics. Zhang [22] employed a 2D continuum vocal fold model to investigate how the location of flow separation influences the phonation set, revealing effects on threshold pressure, frequency, and vibration patterns. In another study, Pelorson et al. [23] employed a boundary layer theory-based approach to investigate the effect of dynamically moving flow separation, finding that it decreases the fundamental frequency and MFDR compared to fixed flow separation. However, a limitation of these studies is that zero pressure was assumed after flow separation, neglecting the effect of negative pressure.

A few more recent studies have explored the potential effects of FSV_NP_ on vocal fold dynamics. Pirnia et al. [24] experimentally investigated the steady state response of cantilevered plates when subjected to tangential advection of periodic generated vortex rings. The velocity field was measured by a particle image velocimetry (PIV) system while the pressure field and the plate energy were calculated using the Poisson pressure equation and Kirchhoff–Love plate theory, respectively. The results were applied to vocal fold vibration by comparing it to a plate with similar non-dimensioned mass and stiffness parameters. They estimated that the ratio of energy transfer due to vortex loading to total aerodynamic energy transfer was negligible. However, modeling the vocal fold structure as a plate is a great simplification of the geometry and the boundary conditions. Moreover, due to experimental limitations, the modulus of elasticity of the plate was as high as 19.9 kPa, which was much larger than the transverse Young’s modulus of the vocal fold reported in the literature [25,26]. Farahani and Zhang [27] employed a 2D computational model of the vocal fold to explore the impact of FSV_NP_ on sound production. They utilized the Bernoulli equation to calculate intraglottal pressures; however, when the glottis was divergent, they introduced a sinusoidal spatial distribution of negative pressure on the vocal fold surface between the flow separation location and the superior edge. The spatial mean value of the applied negative pressure was up to 0.15 times the subglottal pressure, which was estimated from an experimental measurement. They observed a 12.5% increase in MFDR and a 1 dB increase in sound intensity.

The complete understanding of how flow separation and the resulting pressure changes affect voice production remains elusive. In the current study, we aim to quantify the effects of FSV_NP_ on vocal fold vibration by employing an innovative modeling approach. Specifically, we coupled a three-mass vocal fold model with a Navier–Stokes equation-based flow model to simulate fully coupled FSI during vocal fold vibration. Leveraging the Navier–Stokes equation, our FSI model provides high-resolution dynamic pressure solutions on vocal fold surfaces throughout the vibration cycle. To isolate the effect of FSV_NP_ during glottis closing, we created a comparative model that mirrors the original FSI setup, with the only exception of removing the negative pressure loading on the vocal folds when the glottis is in divergent shape. This approach differs from the method in Farahani and Zhang [27], where the treatment of flow separation location, intraglottal negative pressure values and spatial destitution of the pressure was simplified. In our approach, the removed negative pressure in the comparative model is accurately calculated from the Navier–Stokes equations and includes precise spatial and temporal variations. By comparing the simulation results from the original FSI model and the comparative model, we aim to examine the effect of FSV_NP_ on comprehensive vocal parameters, including glottal flow rate, vocal fold vibratory dynamics, and aerodynamic energy transfer. We hypothesize that FSV_NP_ can enhance vocal fold vibration by increasing aeroelastic energy transfer during vibration.

## 2. Materials and Methods

### 2.1. Computational Methodology

The aerodynamics of the glottal flow is numerically simulated using a hydrodynamic/acoustic splitting method [28,29]. The method was developed for simulating low-Mach number flow dynamics to avoid the high computational cost of full compressible flow simulations. Our previous work verified this method for simulating glottal aerodynamics and vocal tract acoustics [30]. The results showed that the splitting method showed good agreement with the compressible flow simulation for low-Mach number internal flow problems with both velocity and pressure boundary conditions. In this method, the flow variables are divided into perturbed and incompressible components: $v=v^{\prime }+V$ and $p=p^{\prime }+P$, where v, $v^{\prime }$, and V represent the total, the perturbed, and the incompressible components of the velocity, respectively. p, $p^{\prime }$, and P represent the pressure’s total, perturbed, and incompressible components. The incompressible components are calculated from the unsteady, viscous, incompressible Navier–Stokes equations:

$$\frac{\partial V_{i}}{\partial x_{i}}=0$$

$$\frac{\partial V_{i}}{\partial t}+\frac{\partial V_{i}V_{j}}{\partial x_{j}}=-\frac{1}{\rho }\frac{\partial P}{\partial x_{i}}+\upsilon \frac{\partial^{2}V_{i}}{\partial x_{j}\partial x_{j}}$$

where ρ and υ are the flow density and the kinematic viscosity of the incompressible flow, respectively. 

The perturbed components are calculated from the linearized perturbed compressible equations (LPCE):

$$\frac{\partial {v^{\prime }}_{j}}{\partial t}+\frac{\partial {v^{\prime }}_{i}V_{i}}{\partial x_{j}}+\frac{1}{\rho }\frac{\partial p^{\prime }}{\partial x_{j}}=0$$

$$\frac{\partial p^{\prime }}{\partial t}+V_{i}\frac{\partial p^{\prime }}{\partial x_{i}}+\gamma P\frac{\partial {v^{\prime }}_{i}}{\partial x_{i}}+{v^{\prime }}_{i}\frac{\partial P}{\partial x_{i}}=-(\frac{\partial P}{\partial t}+V_{i}\frac{\partial P}{\partial x_{i}})$$

where γ is the ratio of the specific heats. When solving the LPCE, the values of the incompressible variables are obtained from the incompressible N-S equation calculation. To resolve the moving geometries, a sharp-interface immersed boundary method based on the ghost-cell approach is employed for treating boundary conditions. The incompressible flow solver is described in more detail in [31], while the compressible flow solver is detailed in [28].

**Figure 1:**
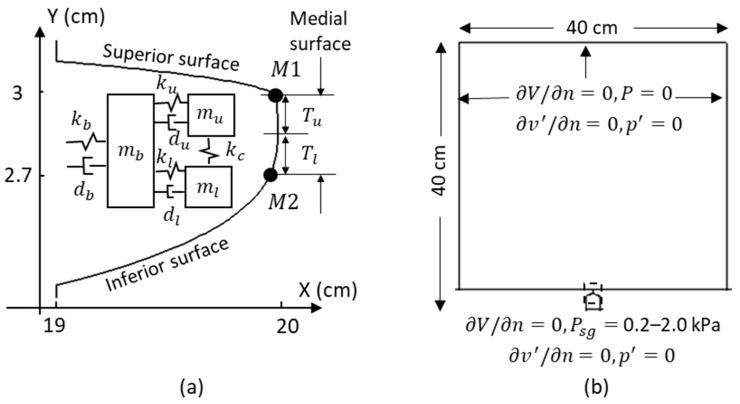
Simulation setup. (**a**) The schematic of the three-mass model of the vocal fold. (**b**) Flow domain and boundary condition. The dashed square highlights the glottal region.

Figure 1a illustrates the schematic of the three-mass model of the vocal fold. In this model, the body-cover structure is represented by three lumped masses connected through springs and dampers [32]. The model only considers the lateral motion of the vocal fold. The equations of the motion of the three masses are

$$m_{u}{\ddot{x}}_{u}=-k_{u}\left[\left(x_{u}-x_{b}\right)+\eta_{u}{\left(x_{u}-x_{b}\right)}^{3}\right]-d_{u}({\dot{x}}_{u}-{\dot{x}}_{b})+k_{c}\left(x_{l}-x_{u}\right)+{Fex}_{u}$$

$$m_{l}{\ddot{x}}_{l}=-k_{l}\left[\left(x_{l}-x_{b}\right)+\eta_{l}{\left(x_{l}-x_{b}\right)}^{3}\right]-d_{l}\left({\dot{x}}_{l}-{\dot{x}}_{b}\right)-k_{c}\left(x_{l}-x_{u}\right)+{Fex}_{l}$$

$$m_{b}{\ddot{x}}_{b}=-k_{b}\left[x_{b}+\eta_{b}{x_{b}}^{3}\right]-d_{b}{\dot{x}}_{b}+k_{u}\left[x_{u}-x_{b}+\eta_{u}{\left(x_{u}-x_{b}\right)}^{3}\right]+d_{u}\left({\dot{x}}_{u}-{\dot{x}}_{b}\right)+k_{l}\left[x_{l}-x_{b}+\eta_{l}{\left(x_{l}-x_{b}\right)}^{3}\right]+d_{l}({\dot{x}}_{l}-{\dot{x}}_{b})$$

where the subscripts u and l represent the upper and lower portions of the cover layer, respectively, and b represents the body layer. $x_{s}$, ${\dot{x}}_{s}$, ${\ddot{x}}_{s}$ (s=u,l,b) represent the displacement, velocity, and acceleration of the masses. $m_{s}$, $k_{s}$, $d_{s}$ (s=u,l,b) are the mass, stiffness, and damping coefficients. $\eta_{s}$ (s=u,l,b) are the nonlinear spring coefficients, and were set to 100 in our simulations. $k_{c}$ is the stiffness of the spring connecting the upper and lower masses. ${Fex}_{s}$ (s=u,l) represent the external force applied on the masses in the lateral direction. $d_{s}$ were calculated as $d_{s}=2\xi_{s}\sqrt{m_{s}k_{s}}$, where $\xi_{u}=\xi_{l}=0.4$ and $\xi_{b}=0.2$ [32].

The vocal fold profile was reconstructed from CT scans of the larynx of a 30-year-old male (Figure 1a) [30]. The profile was represented by one hundred marker points, through which the three-mass model and flow model were coupled. As depicted in Figure 1a, *M*1 (y = 3 cm) and *M*2 (y = 2.7 cm) are the two marker points representing the locations of the upper and lower masses, respectively. At each time step, the velocity and displacement of *M*1 and *M*2 are updated from the values of ${\dot{x}}_{u}$ and ${\dot{x}}_{l}$ in the three-mass model. The velocity and displacement of other marker points are updated using linear interpolations. The flow solver is marched by one time step with the updated vocal fold surface velocity and displacement. Then, the pressure loading on the upper (from y = 2.85 cm to y = 3 cm) and lower (from y = 2.7 cm to y = 2.85 cm) halves of the medial vocal fold surface are integrated to obtain the external forces (${Fex}_{u}$ and ${Fex}_{l}$) acting on the upper and lower masses. Finally, with the updated external forces, the three-mass model is marched by one time step.

In the simulation group where FSV_NP_ was eliminated, an artificial treatment was introduced in the coupling process. Whenever the divergent angle of the two vocal folds surpassed 5 degrees, any negative pressures on the vocal fold surfaces were set to zero when computing the external forces (${Fex}_{u}$ and ${Fex}_{l}$). This process eliminated the influence of negative pressures on the vocal fold dynamics due to flow separation. Nonetheless, the remaining steps of the coupling process remained the same as described earlier.

### 2.2. Simulation Setup

The simulation setup generally follows our previous work [30] metero and a comprehensive validation of the simulation setup was provided in [30]. The parameters of the three-mass model are provided in Table 1. Most of these values are from Story and Titze [32], except for the upper mass, which was increased from 0.01 g to 0.04 g. Previous simulations [30] showed that when the upper and lower masses were of equal value (0.01 g), the model generated small glottal angles (12 degrees between the two vocal folds) that are not conducive to generating flow separations. In addition, excised canine larynges experiments measured maximum divergent glottal angles of 37 and 51 degrees at subglottal pressures of 1.2 and 1.8 kPa, respectively [33]. Based on these observations, we increased the upper mass to achieve larger glottal angles for generating flow separations. With this adjustment, the model generates the maximum divergent angle of 48 and 60 degrees at subglottal pressures of 1.2 and 1.8 kPa, respectively.

Figure 1b depicts the airway configuration. The glottal region is highlighted using a dashed square. Downstream of the glottis, an open environment is represented by a large box measuring 37 cm × 40 cm. The configuration was discretized using 256 × 256 non-uniform Cartesian grids. The vocal fold region (19 ≤ X ≤ 21 cm, 2.17 ≤ Y ≤ 3.17 cm) had the highest grid resolution, with 128 × 98 non-uniform Cartesian grids. The grid density in the vocal fold region was the same as that used in [30], who performed a grid-independent study on a similar configuration.

In the incompressible flow solver, a subglottal pressure was imposed at the glottis inlet, with values ranging from 0.2 to 2 kPa. A zero gauge pressure was applied at the exit of the far-field domain. Non-slip, non-penetration boundary conditions were imposed on the wall of the vocal tract and vocal fold. The incompressible air was assumed to have a density of 1.145 kg/m^3^ and a kinematic viscosity of 1.65 × 10^−5^ m^2^/s, corresponding to 35 °C. The LPCE solver used a hard-wall boundary condition for the vocal fold walls ($\frac{\partial p^{\prime }}{\partial n}=0$, $v^{\prime }\cdot \hat{n}=0$, where $\hat{n}$ denotes the face normal). A buffer zone was incorporated to eliminate acoustic reflections at the inlet of the subglottal tract and the exit of the far-field domain. During vibrations, the vocal fold contact was modeled by enforcing a 0.16 mm minimum gap between the folds until the aerodynamic force was sufficient to separate them. This gap size was approximately 6% of the maximum gap observed at 1.2 kPa subglottal pressure.

## 3. Results and Discussion

### 3.1. Flow Dynamics

**Figure 2:**
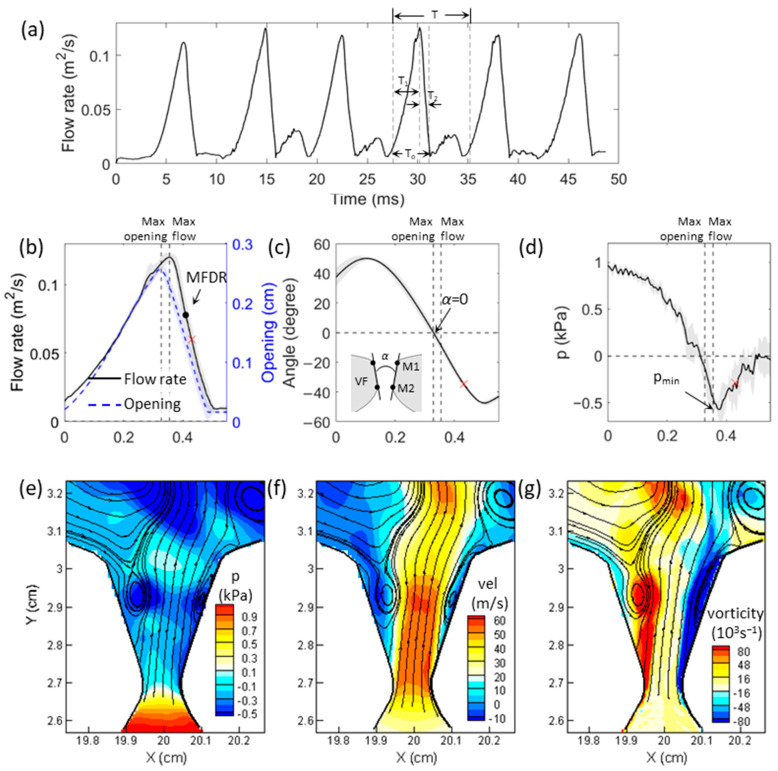
Glottal flow parameters and flow field contours in the baseline configuration at 1.2 kPa subglottal pressure. (**a**) Time history of the glottal flow. Phase averaged (**b**) flow rate and glottal opening, (**c**) glottal angle, and (**d**) mean pressure of the medial surface of the vocal folds, respectively. MFDR instant is denoted in (**b**). (**e**–**g**) Show intraglottal pressure, vertical velocity, and vorticity field, respectively, with flow streamlines superimposed. The phase time corresponding to (**e**–**g**) is denoted as a cross ‘x’ in (**b**–**d**). In (**a**), T is the period of the vibration; T_o_ is the duration of open glottis; T_1_ and T_2_ correspond to the time duration of flow acceleration and deceleration, respectively. See text for a detailed description of the figures.

A series of FSI simulations were conducted by varying the subglottal pressure from 0.2 to 2 kPa in increments of 0.2 kPa. These cases are referred to as baseline cases. Sustained vibrations were observed when the subglottal pressure was above 0.4 kPa. The resulting fundamental frequency ranged from 101 to 144 Hz, glottal opening ranged from 1.2 to 3.5 mm, maximal divergent angle ranged from 12 to 62 degrees, open quotient mostly ranged from 0.51 to 0.53, and skewness quotient was around 2.5. The glottal opening was determined by measuring the minimum distance between the upper and lower masses. The glottal angle (α) was defined as the angle between the two vocal fold medial surfaces (Figure 2c) and was calculated using the positions of the *M*1 and *M*2 markers points:

$$\alpha =\mathrm{atan}\left(\frac{{dx}_{M1}-{dx}_{M2}}{{dy}_{M1,\;M2}}\right)$$

where ${dx}_{M1}$ and ${dx}_{M2}$ are the lateral distances between the pair of *M*1 and *M*2, respectively, and ${dy}_{M1,\;M2}$ is the vertical distance between *M*1 and *M*2, which is the same for both sides of the vocal folds. The open quotient was defined as a ratio of the open glottis duration (T_o_) to the vibration period (T). The skewness quotient was the duration ratio of the flow acceleration (T_1_) to flow deceleration (T_2_). T, T_o_, T_1_ and T_2_ are denoted in Figure 2a in one of the vibration cycles. Unless otherwise noted, the data reported in the results section averaged over four consecutive sustained cycles. The fundamental frequency, open quotient, and skewness quotient fell within the expected physiological range [34]. For the glottal opening and the maximal divergent angle, the value under high subglottal pressure is higher than what is typically measured or employed in other studies. For instance, the maximal divergent angle measured in an excised canine larynx was reported to be 51 degrees at 1.8 kPa subglottal pressure [33]. The divergent angle of up to 40 degrees was typically employed in studies involving synthetic vocal folds [9,13] or simulations [4,6,35]. A glottal width of 3 mm was utilized in the numerical study of intraglottal vortices [35]. However, it is worth noting that the subglottal pressures in those referenced studies were also lower. For instance, in [13], subglottal pressure ranged from 0.3 to 1.48 kPa; in [9], [6], [4], and [35], it was 0.5 kPa, 0.35 kPa, 0.59 kPa, and 0.3 kPa, respectively. The higher subglottal pressure employed in our study could potentially have contributed to the large glottal angle and opening. A larger divergent angle has been shown to shift the flow separation location upstream [10,22,36]. However, as reported in [36], when the divergent angle was 40 degrees, the flow separation location was close to the glottis entrance, with the reverse flow just past the separation points. This flow separation pattern is similar to what we observed in our current study (Figure 2). Therefore, we consider the glottal opening and divergent angle we observed to be within the reasonable range, and though the divergent angle may exceed 40 degrees under high-pressure conditions in our study, it might affect the flow separation location only in a very limited range near the glottis entrance.

The baseline cases displayed consistent vibratory dynamics and flow patterns. To illustrate this, we present the 1.2 kPa subglottal pressure case data, which is representative of all cases, in Figure 2. Figure 2a depicts the time history of the flow rate, demonstrating sustained vibration. Figure 2b–d shows the phase-averaged data of flow rate and glottal opening, glottal angle, and spatial-average flow pressure on the vocal fold medial surface, respectively. Phase time is normalized to 0–1, with 0 indicating the beginning of the glottal opening. This figure focuses on the duration of the opening and closing phases (from 0 to 0.55). The flow rate was calculated at the glottal exit. A positive value of the glottal angle indicates a convergent shape, and a negative value indicates a divergent glottis. The average flow pressure on the vocal fold medial surface was calculated by averaging the external forces on the upper and lower masses and dividing it by the thickness of the vocal fold medial surface. The lines depict the phase-averaged values, while the width of the shaded region shows the standard deviation. The two vertical dashed lines indicate the moments of maximum glottis opening and maximum flow rate, and the horizontal dashed lines indicate zero glottal angle and zero vocal fold surface pressure, respectively.

The results show that during the initial phase of the glottal opening, a large positive (convergent) angle was present due to the earlier opening of the inferior edge. This is attributed to the propagation of a mucosal wave on the vocal fold surface. As the flow rate increased, the convergent angle also increased until it reached its maximum. The surface pressure decreased rapidly during the entire opening phase. At the point of maximum glottal opening, the angle reduced to zero, and the surface pressure also dropped to nearly zero. As the glottis began to close, the angle became negative, indicating a divergent glottal shape. The divergent angle continued to increase during closing and reached its maximum towards the end of the closing phase. The surface pressure was negative throughout the closing phase, dropping to its lowest point (−0.57 kPa when Phase = 0.37) shortly before the MFDR occurred. There was a small phase difference between the maximum glottis opening and maximum flow rate, likely due to the small inertance in the downstream box. The most negative intraglottal pressure corresponds to 48% subglottal pressure, which is in line with observations from previous studies (−0.5 to −0.2 times subglottal pressure) [9,12,13,14,15]. The mean negative pressure during the closing phase was approximately −0.33 kPa, equivalent to 28% of the subglottal pressure.

In Figure 2e–g, the contours of flow pressure, the vertical component of flow velocity, and vorticity inside the glottis at a representative time instant during glottal closing (Phase = 0.43) are shown. The phase time corresponding to Figure 2e–g is denoted as cross in Figure 2b–d, which locates around the mid-closing phase. Additionally, flow streamlines are superimposed to illustrate the separated flow patterns. A recirculation zone is formed between the glottal jet and each medial wall, characterized by flow entering from above the glottis and forming FSV. The locations of the lowest pressure and the strongest vorticity correspond to the FSV location. The calculated vorticity exhibits a magnitude level similar to that reported in [8].

Overall, the baseline simulations demonstrate flow and vibration dynamics that are in line with physiological observations and quantities. During the glottis closing, a recirculation zone is formed between the glottal jet and each vocal fold wall. This leads to the generation of local negative pressures and influences the adjacent wall pressures. Specifically, for the representative subglottal pressure of 1.2 kPa, the resultant wall pressure on the medial surface of the vocal fold during the closing phase reaches a minimum value of −0.57 kPa and a mean value of −0.33 kPa, corresponding to 48% and 28% of the subglottal pressure, respectively.

### 3.2. Effects of Eliminating FSV_NP_

In this section, we compare the baseline results with a series of simulations under the same conditions, except that the negative pressures applied on the medial wall during the closing phase are set to be atmospheric. This process (referred to as (-) FSV_NP_) aims to eliminate the FSV_NP_ contribution to the mechanism of the fold vibrations.

**Figure 3:**
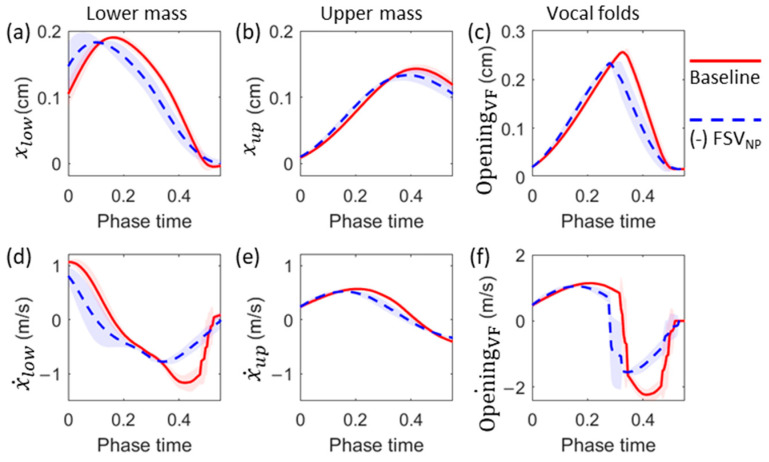
Comparison of the vibration parameters between the baseline and (-) FSV_NP_ cases at a representative subglottal pressure of 1.2 kPa. The lines show the phase-averaged values, and the shaded area indicates the standard deviation. (**a**,**b**) Displacement of the lower mass and upper mass, respectively. (**c**) Glottal opening. (**d**,**e**) The velocity of the lower mass and upper mass, respectively. (**f**) Time-derivative of the glottal opening.

Figure 3a–c compares the displacement of the masses (M1 and M2) and glottal opening with their corresponding time derivatives shown in Figure 3d–f for the representative subglottal pressure of 1.2 kPa. Due to left-right symmetry, only one vocal fold was studied (Figure 3a,b,d,e). The glottal opening (Figure 3c) was determined as the minimum distance between the two sides of the masses. Figure 3f is the derivative of the glottal opening, representing the speed of the vocal fold displacement. In the closing phase, it is referred to as the closing speed. Overall, the (-) FSV_NP_ case demonstrates smaller vibration amplitudes and closing speeds than the baseline case. Quantitatively, the vibration amplitude of the lower and upper masses in the (-) FSV_NP_ case was 3.8% and 6.8% lower than those in the baseline case, respectively. The maximum glottis opening in the (-) FSV_NP_ case was 8.8% lower than the baseline. The maximum closing speed of the upper and lower mass in the (-) FSV_NP_ case was 26.3% and 33.3% smaller than those in the baseline case. For the upper mass, due to the phase delay between the two masses, the maximum closing speed occurred in the closed phase (thus not shown). The maximum glottal closing speed in the (-) FSV_NP_ case was 30.5% smaller than in the baseline case. These results suggest that the FSV_NP_ played an essential role in promoting vocal fold vibration and facilitating glottis closing, likely because the negative pressures pull the vocal fold to close, resulting in additional energy transfer to the vocal fold.

**Figure 4:**
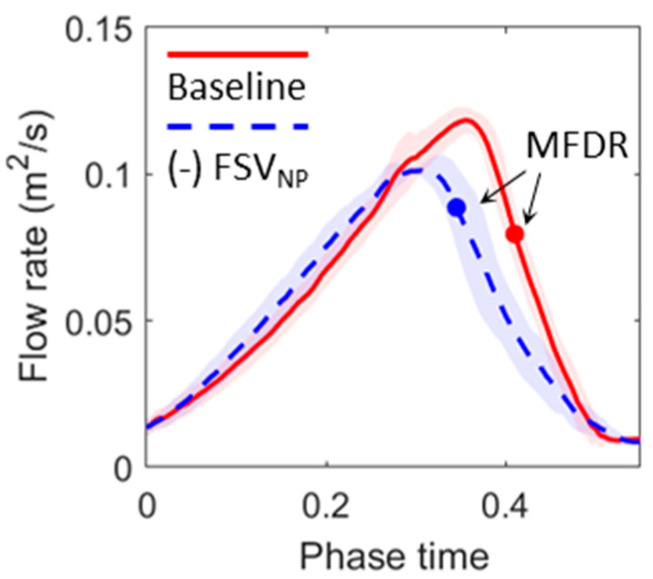
Comparison of the flow rate between the baseline and (-) FSV_NP_ cases at a representative subglottal pressure of 1.2 kPa. The line plot shows the phase-averaged value, and the shaded area indicates the standard deviation. MFDR instants are denoted.

Figure 4 compares the phase-averaged flow rate between the baseline and (-) FSV_NP_ cases at 1.2 kPa subglottal pressure. The (-) FSV_NP_ case showed a lower flow rate and slower deceleration, leading to smaller MFDR and flow skewness values. Quantitatively, the MFDR in the (-) FSV_NP_ case was 80 m^2^/s^2^, 34% lower than the value in the baseline case (120.7 m^2^/s^2^). The flow skewness quotient was 1.64, 41% lower than the value in the baseline case (2.8). These observations are consistent with the reduced glottal opening and closing speed in the (-) FSV_NP_ case.

**Figure 5:**
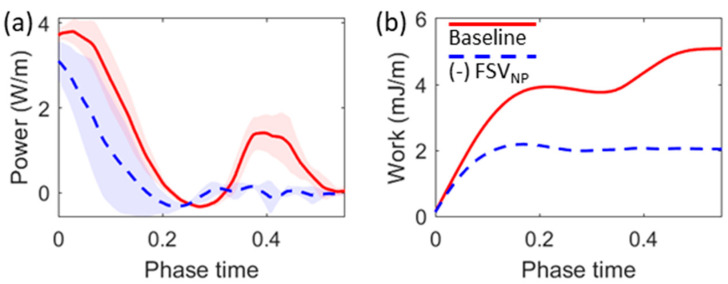
Comparison of (**a**) power transfer from the airflow to the vocal fold and (**b**) the total flow work over the medial wall during opening and closing phases between the baseline and (-) FSV_NP_ cases. The line plot in (**a**) shows the phase averaged value while the width of the shaded area indicates the standard deviation. In (**b**), the work is shown for one vibration cycle to avoid the accumulation effect due to the integration over time.

To investigate the aeroelastic energy transfer, we computed the power transfer from the airflow to the vocal fold and the total flow work over the vocal fold medial surface during the opening and closing of the fold vibrations. The power transfer was computed by multiplying the flow pressure by the normal component of the velocity vectors and integrating over the vocal fold medial surface. The flow work was then obtained by integrating the power values over time. The overall energy balance did not consider viscous force because their contribution is about two orders of magnitude smaller than the aerodynamic pressure [37]. Figure 5 compares the time history of power transfer and flow work when the glottis was open between the (-) FSV_NP_ and baseline cases. The power plot shows the phase-averaged values as curves, with the shades representing the standard deviation. The work plot shows data from one vibration cycle to avoid the accumulation effect due to the integration over time. In the baseline case, a distinct peak in power transfer was observed around the time when the maximum FSV_NP_ (Figure 2d) and maximum glottis closing speed (Figure 3f) occurred. The flow work plot shows that in the baseline case, the flow work continuously increased during most of the closing phase, while, in the (-) FSV_NP_ case, it remained almost constant during glottis closing. These results support the notion that FSV_NP_ generated additional energy transfer from the airflow to the fold during the closing phase. 

**Figure 6:**
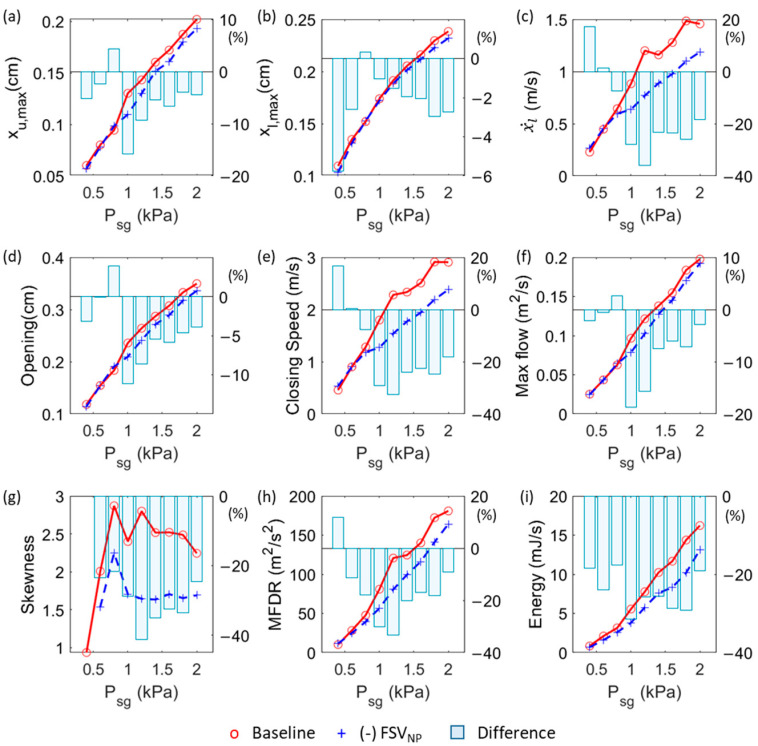
Comparison of important vibration and flow parameters between the baseline and (-) FSV_NP_ cases over the range of simulated subglottal pressures. (**a**) Maximum opening of the upper mass. (**b**) Maximum opening of the lower mass. (**c**) The maximum closing speed of the lower mass. (**d**) Glottal opening. (**e**) Vocal fold closing speed. (**f**) The maximum glottal flow. (**g**) Skewness quotient. (**h**) Maximum flow declination rate (MFDR). (**i**) Total energy transfer from the airflow to vocal fold tissue during one vibration cycle.

In Figure 6, we compare key vibration and flow parameters between the baseline and (-) FSV_NP_ cases over the range of simulated subglottal pressures. The parameters examined include the maximum opening of the upper and lower masses (a,b), maximum closing speed of the lower mass (c), maximum glottis opening (d), maximum glottis closing speed (e), maximum flow rate (f), flow skewness quotient (g), MFDR (h), and energy transfer in one vibration cycle (i). The percentage difference between the two cases is represented by the bars in the figures. The results indicate that removing FSV_NP_ had a more pronounced effect when the subglottal pressure was above 1 kPa. These effects were consistent across this pressure range, causing reduced vibration amplitude, closing speed, flow rate, flow skewness, MFDR, and energy transfer. Quantitatively, the effects on vibration amplitude and flow rate were up to 20%, while those on closing speed, flow skewness quotient, MFDR, and energy transfer were more significant, reaching up to 40%. The most notable effects were observed in the 1–1.2 kPa subglottal pressure range. No consistent effects were observed below a subglottal pressure of 1 kPa. Moreover, some relative errors may appear large at low subglottal pressures due to small values in the baseline case (e.g., Figure 6b,c,e at a subglottal pressure of 0.4 kPa).

In our study, removing FSV_NP_ at a subglottal pressure of 1.2 kPa, corresponding to approximately 28% of the subglottal pressure, resulted in a 34% reduction in MFDR compared to the baseline. Farahani and Zhang [27] reported similar findings, showing that applying additional negative pressures downstream of the flow separation with a mean value of approximately 15% of subglottal pressure resulted in a 12.5% increase in MFDR. They estimated that a 12.5% increase in MFDR resulted in a 1 dB change in SPL. Using the same method, we estimated that the 34% decrease in MFDR would result in a 2.4 dB change in SPL.

These results suggested that FSV_NP_ enhances vocal fold vibrations and sound intensity during voice production by promoting greater energy transfer from the airflow to the vocal fold. It is also worth noting that when the FSV_NP_ was removed, complete glottis closure was never achieved at the lowest subglottal pressure of 0.4 kPa, whereas it was achieved in the baseline case. This observation also suggests the importance of FSV_NP_ in facilitating stronger vibrations.

## 4. Conclusions

This study employed a two-dimensional FSI computer model to investigate the effects of FSV_NP_ on vocal fold vibration and flow dynamics during vocal fold vibration. The numerical model integrated a Navier–Stokes equation-based incompressible flow model, a linearized perturbed compressible equation-based acoustic model, and a three-mass vocal fold model. The baseline simulations predicted flow and vibration dynamics that are consistent with physiological observations and quantities. Specifically, FSV formed between the medial wall and the separated glottal flow resulting in an intraglottal pressure that was predominantly negative after the location of flow separation and before gradually recovering to zero at the glottis exit. In a representative subglottal pressure of 1.2 kPa, the mean negative pressure in the closing phase was approximately −0.33 kPa, equivalent to 28% of the subglottal pressure. The maximum FSV_NP_ occurred near the time instant of MFDR and corresponded to the location of the FSV.

To isolate the effects of FSV_NP_, a comparison simulation group was conducted in which negative pressures arising after flow separation were removed in FSI. Notably, the results showed consistent effects above a subglottal pressure of 1 kPa, where removing FSV_NP_ resulted in reduced vibration amplitude, flow rate, closing speed, flow skewness quotient, and MFDR. Quantitatively, the vibration amplitude and flow rate were reduced by up to 20%, and the closing speed, flow skewness quotient, and MFDR were reduced by up to 40%. In energy transfer analysis, the FSV_NP_ generated an additional energy transfer peak from the airflow to the vocal fold during glottis closing, thereby increasing overall energy transfer over a cycle. Quantitatively, removing FSV_NP_ resulted in an energy loss of up to 32%. These findings suggest that FSV_NP_ enhances vocal fold vibration by increasing the aeroelastic energy transfer during vibration. This result emphasizes the significant role of the FSV on the vocal fold dynamics.

It is important to acknowledge the limitations of this study. Firstly, though two-dimensional assumption is very frequently employed in the simulations of the vocal fold vibration and laryngeal flow [38], previous studies have shown that the negative pressure from a model with a constant glottal shape in the anterior–posterior direction could be 16% lower than a model with the glottal opening gradually decreasing to zero towards the two ends [12]. Additionally, the pressure variation in the latter model was largest around the mid-coronal plane. It decreased towards the two ends, with intraglottal pressure always being positive at the two ends [15]. In the current study, only mid-coronal plane parameters were considered by employing a two-dimensional model, which might have overestimated the mean intraglottal negative pressure. Therefore, caution should be taken when generalizing these results to three-dimensional scenarios.

The second limitation is that the three-mass model used in the study has a larger upper mass than the lower mass to achieve the desired glottal divergent angle and flow separation during closing, which is opposite to what is usually seen in in two-mass models, where the lower mass is greater to mimic the characteristics of the body layer [23,39]. Nevertheless, it was not the first time that a larger upper mass was used in a three-mass model [40]. Ultimately, our simulation results indicated that our model parameters predicted reasonable dynamics of vocal fold vibrations, with dynamic parameters that agree with physiological quantities.

Overall, this study contributes to a deeper understanding of the FSV and the associated FSV_NP_’s influence on vocal fold vibration and flow dynamics, which may have implications for improving voice production and treating voice disorders.

## Figures and Tables

**Table 1:** Parameters of the three-mass model of the vocal folds.

	$m_{u}$ (g)	$m_{l}$ (g)	$m_{b}$ (g)	$k_{u}$ (N/m)	$k_{l}$ (N/m)	$k_{b}$ (N/m)	$k_{c}$ (N/m)	$T_{u}$ (cm)	$T_{l}$ (cm)
Value	0.04	0.01	0.05	5	7	100	1	0.15	0.15

## Data Availability

The data presented in this study are available upon request.
